# Modern Vaccination

**Published:** 1901-12-21

**Authors:** 


### MODERN VACCINATION.

At the last meeting of the Royal Medical and Chirurgical Society Dr. Monckton Copeman gave an interesting demonstration on modern methods of vaccination, in the course of which he explained the common belief that calf lymph produced "worse arms" than the method of arm-to-arm vaccination, by showing that culture plates inoculated from calf lymph showed a greater number of colonies of micro-organisms than when inoculated from lymph taken from the human arm. This he had no doubt was due to the greater difficulty in cleansing the skin of the calf as compared with that of the child. The opacity which occurred in stored lymph was due, he said, to the increase in the number of bacteria in the tube. He had, however, been able to obtain inhibition of the growth of these organisms by mixing lymph with 50 per cent. of pure glycerine in water, and he had been able to show by a series of plate cultivations that the number of bacteria steadily diminished, so that at the end of a month no organisms were present on the culture medium. Not only did this method kill all the usual contaminating bacteria, but it would also destroy those of tubercle and erysipelas when these were added experimentally in comparatively large quantities.

Dealing with the relation of vaccinia to small-pox, he stated his belief that the former was an attenuated form of the latter. It was well known, he said, that a calf could seldom be inoculated directly from a case of even confluent small-pox in the human subject, but that if a monkey were inoculated with lymph from such a case good vesicles developed. If lymph were then taken from these vesicles and a calf inoculated with it, poor vesicles were produced, but if a second calf was then inoculated from the first a good crop of vesicles was produced, and if then a child was inoculated with lymph from this calf a typical vaccinia was produced.

